# The inclination of the tibial component has an impact on fracture stability in unicompartmental knee arthroplasty: an artificial bone study

**DOI:** 10.3389/fbioe.2025.1615216

**Published:** 2025-10-14

**Authors:** Mathis Wegner, Maximilian Bettendorff, Malte Bruhn, Jörg Bahr, Jürgen Carstensen, Leonard Siebert, Babak Moradi

**Affiliations:** ^1^ Department of Orthopedics and Trauma Surgery, University Hospital Schleswig-Holstein, Kiel, Germany; ^2^ Department for Materials Sciences, Functional Nanomaterials Chair, Faculty of Engineering, Kiel University, Kiel, Germany

**Keywords:** unicompartmental knee arthroplasty, periprosthetic fracture, tibial component, varuspositioning, PPF

## Abstract

**Background:**

Periprosthetic fractures (PPFs) following unicompartmental knee arthroplasty (UKA) are a significant clinical challenge. Tibial component positioning may influence fracture risk, but the biomechanical effects of varus inclination on fracture loading remain unclear.

**Methods:**

We investigated the effect of tibial component varus inclination on fracture load using the Oxford^®^ Partial Knee implant system, synthetic tibiae and a dynamic loading model. Tibial components were implanted at neutral (0°), 3° and 6° varus angles. Vertical loading was applied until fracture and fracture loads were compared between groups.

**Results:**

A 3° varus position significantly increased fracture load by 34% compared to neutral (p < 0.05). No further statistically significant increase was observed at 6° varus. The dynamic model suggested that the mobile meniscal bearing may contribute to an improved load distribution, thereby increasing fracture resistance.

**Conclusion:**

Slight varus inclination of the tibial component in UKA increases the medial tibial fracture load, potentially reducing the risk of PPF. Our findings highlight the biomechanical advantages of controlled varus positioning and provide insight into optimizing implant alignment.

## 1 Introduction

Unicompartmental knee arthroplasty (UKA) represents a well-established surgical therapeutic option for the treatment of unicompartmental osteoarthritis (OA) of the knee. It is estimated that approximately 50% of the global population will develop end-stage osteoarthritis during their lifetime, which represents a significant epidemiological burden for society (Murphy et al., 2008). In Germany, 137,030 primary knee arthroplasty procedures were performed in 2022, with Total knee arthroplasties (TKA) accounting for 87.1% and UKAs accounting for 12.7% of this total (Endoprothesenregister Deutschland EPRD, 2023). TKA has been demonstrated to significantly improve function and quality of life, without exerting an overall effect on morbidity and mortality (Wilczyński et al., 2024). The rising demand for arthroplasty in a young population, coupled with the increasing number of surgeries being performed, has led to a growing body of research examining the complications and revisions associated with standard cemented total knee arthroplasty (TKA) (Irmola et al., 2021; Prudhon and Verdier, 2017). Despite the invention of cementless fixation systems (Papas et al., 2019; Duffy et al., 1998), a total of 14,379 revision surgeries were performed in Germany in 2023, with aseptic loosening being the predominant cause of revision at 22.8% (Endoprothesenregister Deutschland EPRD, 2023).

The primary indication for UKA is anteromedial osteoarthritis with intact ligaments and a correctable varus deformity. In comparison to TKA, the advantages of UKA over TKA have been the subject of extensive research, and include the preservation of native bone stock, lower blood loss, reduced infection rates, preservation of normal knee kinematics and superior functional outcomes (Clarius et al., 2010; Wood et al., 2024; Burn et al., 2018; Liddle et al., 2015). Nevertheless, the percentage of UKA implantation in primary knee arthroplasties in Germany has decreased from 13.2% in 2021 to 12.7% in 2022 (Endoprothesenregister Deutschland EPRD, 2023; Endoprothesenregister Deutschland EPRD, 2022). The most probable explanation is that joint registries document a higher probability of failure and higher revision rates compared to TKAs (Endoprothesenregister Deutschland EPRD, 2023; Endoprothesenregister Deutschland EPRD, 2022; Willis-Owen et al., 2009). Reasons for UKA revision include aseptic loosening, malalignment, progression of OA, instability, infection, and periprosthetic fracture (PPF) (Wood et al., 2024).

PPF represent a rare yet serious complication in UKA. Most reported at the tibial side, incidences range from 1.2% up to 8% in literature (Endoprothesenregister Deutschland EPRD, 2023; Wood et al., 2024; Burger et al., 2022; Dai et al., 2018; Hiranaka et al., 2020; Yoshida et al., 2013; Mohammad et al., 2023). PPFs are most frequently observed because of patient-specific risk factors, technical errors or lack of precision during surgical procedures (Burger et al., 2022; Binkley et al., 2023). Patient-specific risk factors that favor a higher incidence of PPF are an advanced age, female sex, lower bone mineral density, and specific tibial bone morphology (Wood et al., 2024; Binkley et al., 2023). An under sizing or oversizing of the tibial implant, an extended sagittal saw cut, and a decreased keel-cortex distance and improper alignment of the tibial component have been identified as risk factors for PPF as well (Clarius et al., 2010; Wood et al., 2024; Burger et al., 2022; Mohammad et al., 2023; Suda et al., 2022; Watrinet et al., 2024a; Watrinet et al., 2024b). The incidence of PPF in cemented and cementless UKAs is comparably low. However, excessive compression in combination with an impaction technique may pose an additional risk of PPF in cementless UKAs (Burger et al., 2022).

The alignment of the tibial component in UKAs has been the subject of several studies in current literature. It is believed that the optimal mechanical stress distribution is achieved with a neutral tibial component, as demonstrated in TKAs (Iesaka et al., 2002; Fang et al., 2009). Previous finite element analyses (FEA) on load transfer and stress distribution in UKAs have demonstrated that a varus tilt of 2°–4° of the tibial component in the coronal plane reduces the peak load on the medial tibial cortex and avoids an increase in stress between the keel tip and the medial tibial cortex (Wood et al., 2024; Dai et al., 2018; Fang et al., 2009). These findings have been clinically confirmed (Watrinet et al., 2024a; Watrinet et al., 2024b).

The aim of this study is to assess the impact of the varus angle of the tibial component on the fracture load distribution using the Oxford^®^ mobile partial knee implant system to investigate fracture toughness of the tibia angles between 0° and 9° were used. Additionally, we used a simple dynamic model accounting for friction, band stretching and initial force to elucidate the possible mechanism for fracture toughness increase and calculate an optimal post-UKA tibio-femoral alignment in the coronal plane.

## 2 Methods

### 2.1 Ethics approval

The experimental study was approved by the Institutional Review Board of the University Medical Center Schleswig-Holstein (D 628/23) and was conducted in accordance with the Declaration of Helsinki.

### 2.2 Experimental procedure

A *size A* tibial component of the Oxford cementless partial knee (Zimmer Biomet Holdings, Warsaw, Indiana, United States of America) was implanted into 31 specimens of polyurethane right tibiae (Synbone AG, Switzerland). Inclination angles were set at 0°, 3°, 6° and 9° varus. The slope angle was maintained at 0°. Mechanical testing was performed in a compression testing machine under standardized conditions, employing a single-cycle load-to-failure protocol, at a displacement rate of 10 mm/min, while the applied force and displacement were recorded. Two sets of tests were performed: first forces were applied not perpendicular to the implant, but parallel to the tibial shaft axis. Then another set with the load perpendicular to the implant surface was made. A mobile meniscal bearing (Zimmer Biomet Holdings, Warsaw, Indiana, United States of America) was used to ensure a realistic load transmission and distribution from the mechanical testing machine onto the implant. Forces were applied until a fracture of the medial tibial plateau occurred.

### 2.3 Experimental set-up

**FIGURE 1:**
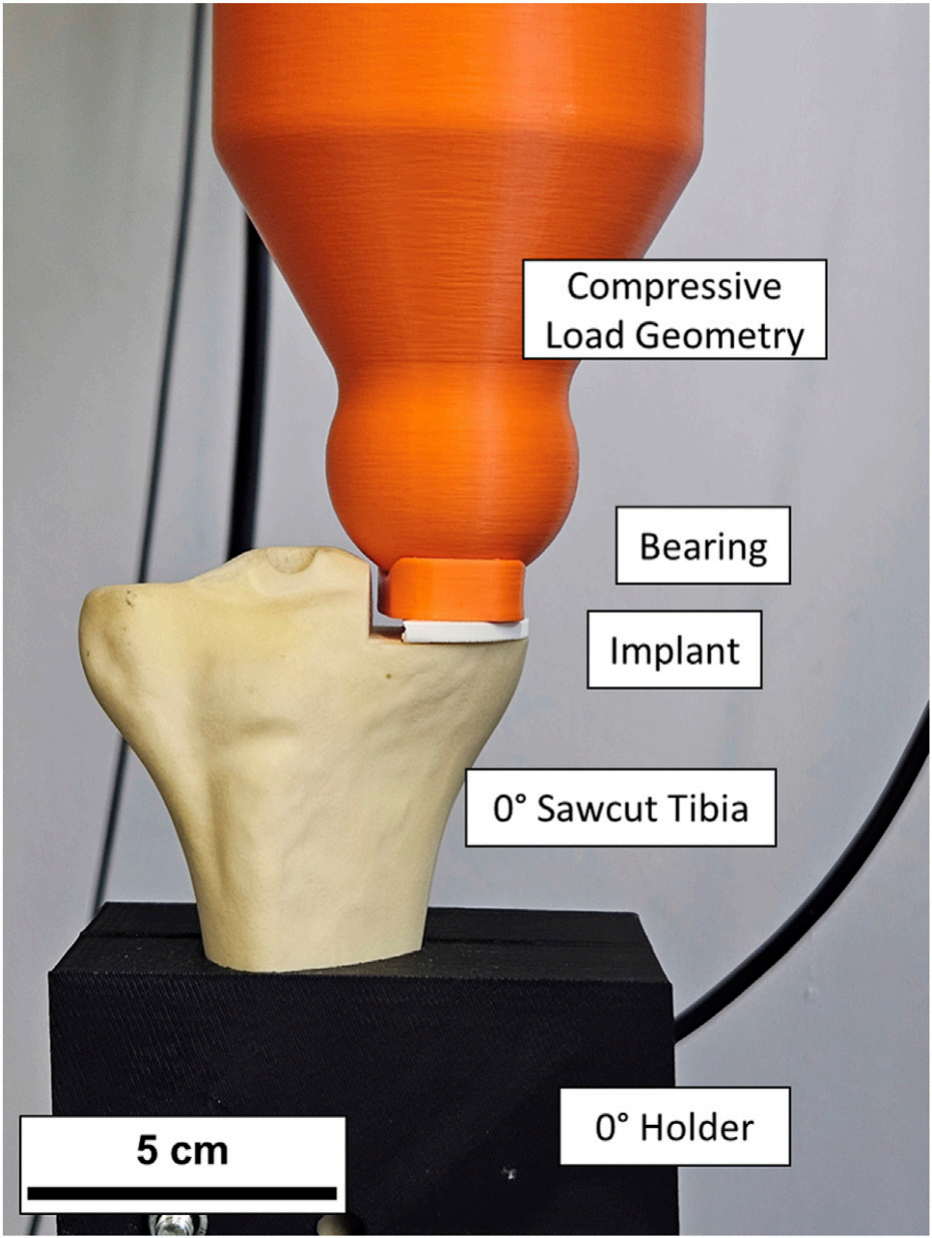
Sawcut tibia bone with implant and bearing in the compression test apparatus. The implant and meniscal bearing in this instance were 3D printed for setup validation and for the real tests replaced with originals.

The distal tibia was removed leaving 20 cm distal to the tibial plateau. The tibiae models were 3D-scanned on a Keyence VL-500 and digitized to place the tibial implant reproducibly (cf. Supporting Figure 1) and to 3D-print fitting holders on a Prusa MK3 filament printer. Using these holders, the saw cuts for the preparation of the tibial plateau were made by a computer numerical control (CNC) milling machine ensuring high precision and reproducibility (cf. Supporting Figure 2). For mechanical testing, suitable mounts were 3D-printed, which hold the specimens in an angle that was equalizing the inclination for perpendicular testing (Figure 1). One additional test group, with a sawcut of 3° varus, was prepared and mounted an angle of 0° for testing load transmission parallel to the tibial shaft axis. All tibiae were loaded under standardized conditions in a compression testing machine.

**FIGURE 2:**
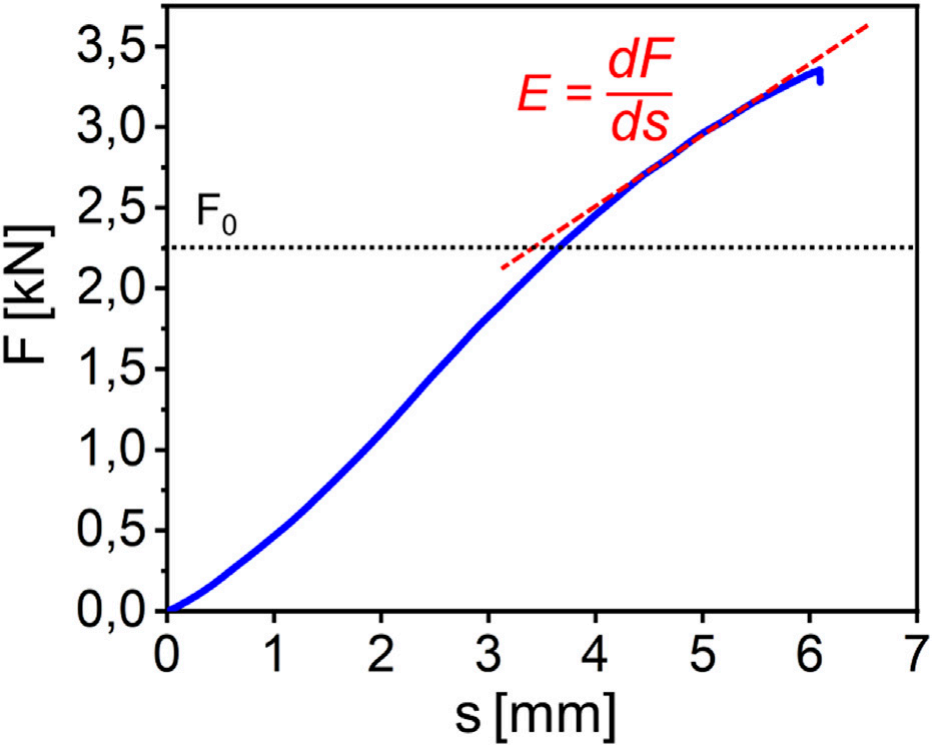
Exemplary force displacement curve for a tibia loaded until fracture. Two distinct linear regions can be identified. The slope of the second region is defined as E. The transition point between first and second region is defined as F_0_.

### 2.4 Statistical analyses

The statistical analysis was conducted using a custom python-script and Microsoft Excel (Version 16.91, Microsoft Corporation, Redmond, Washington, USA). The means and standard deviations were calculated. The Kolmogorov–Smirnov test confirmed a normal distribution. The Student's t-test was applied for statistical comparison. A significance threshold of p ≤ 0.05 was set, and results with p-values equal to or less than this threshold were considered statistically significant.

## 3 Results

5 Groups of artificial bones with a different varus inclination or a different angle of force application were tested. The resulting constant deformation velocity force-displacement curves (exemplary depicted in Figure 2) show a typical elastic regime followed by a regime where irreversible deformation and fracture occurs.

When applying forces perpendicular to the implant, a varus inclination resulted in an increased fracture load. For 0° varus the mean fracture load was 2.54 kN ± 0.18 kN, for 3° varus 3.34 kN ± 0.49 kN, for 6° varus 3.36 kN ± 0.38 kN and for 9° varus 3.79 kN ± 0.41 kN. Statistically significant differences were demonstrated by each of the experimental groups in comparison to the 0° varus control group (For 3° p = 0.0089; for 6° p = 0.0001; for 9° p = 0.0001).

**FIGURE 3:**
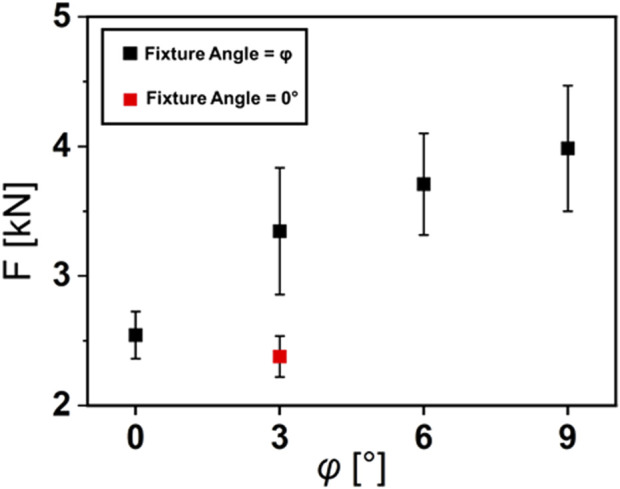
Fracture Loads depending on the varus inclination φ of the tibial saw cuts. In the test groups (black rectangles), forces where applied perpendicular to the implant. In the red test group, forces where applied parallel to the tibial shaft axis.

Differences between the different inclination angles were not statistically significant (for 3° and 6°, p = 0.23; for 6° and 9°, p = 0.56; for 3° and 9°, p = 0.053). When forces were applied parallel to the tibial shaft axis on the implant at a 3° varus inclination, the mean fracture load was 2.37 kN ± 0.15 kN. In this case no significant difference in fracture load was observed compared to the control group. All fracture loads depending on the inclination of the tibial saw cuts can be seen in Figure 3.

**FIGURE 4:**
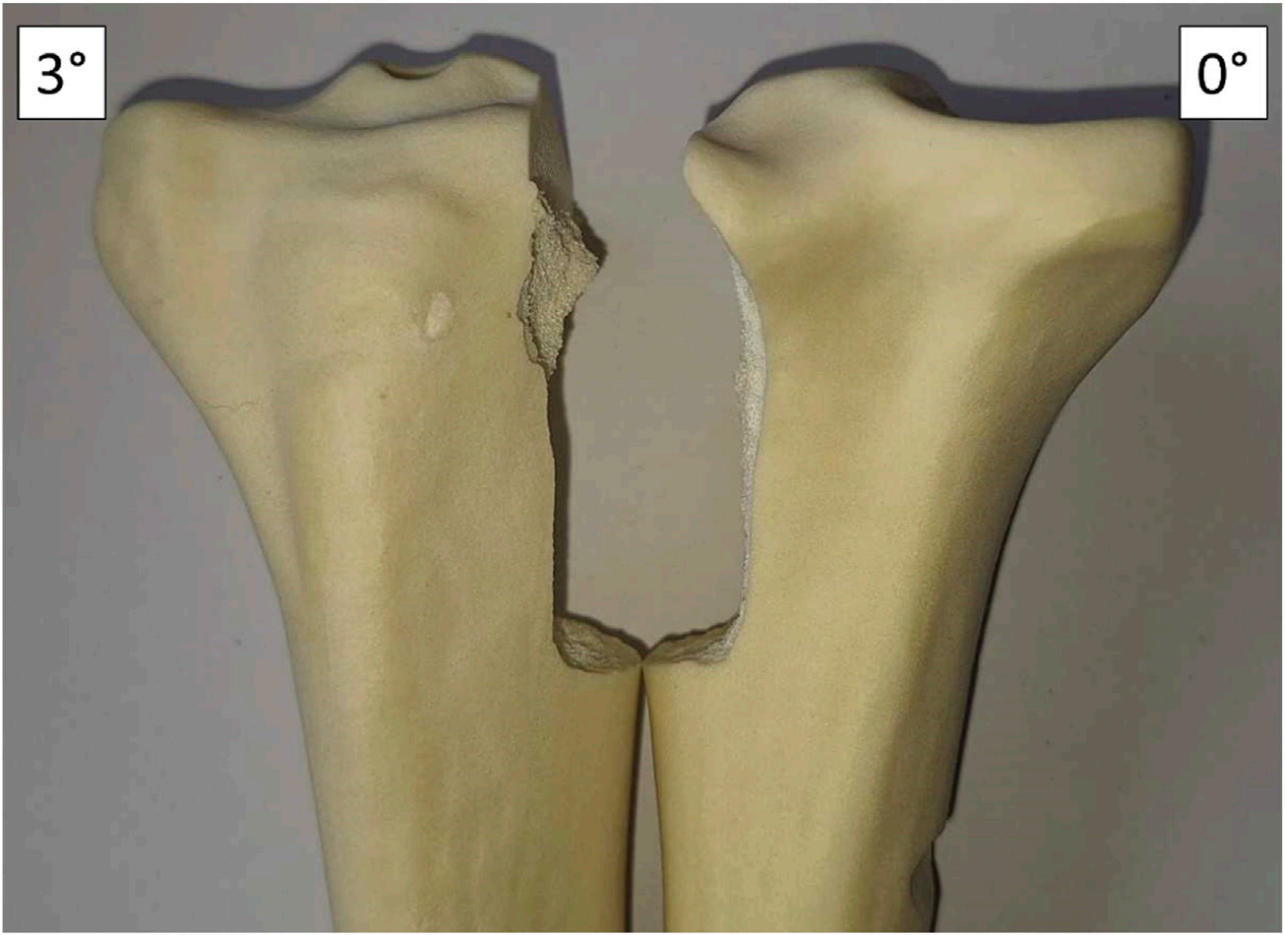
Fracture profiles of the tibiae with 3° and 0° varus inclination. At 3° inclination, the fracture becomes more complex and the fracture area is increased.

Additionally, the load application in 0° always resulted in a straight fracture profile, while the application of force perpendicular to the implant surface at *φ* > 3° always resulted in a more complex fracture profile and a significantly increased fracture surface area (Figure 4).

A decisive observation is that with increase in varus inclination also the variance of the fracture load between the specimens increased. Plotting the variance over the varus inclination angle *φ* results in a straight line which is the clearest hint for a direct correlation.

The fracture profiles of the tibiae showed a straight cut for all samples tested parallel to the tibial shaft axis and a larger, more complex fracture profile when tested perpendicular to the plateau surface (Figure 3).

## 4 Discussion

The principal outcome of our experimental investigation was that the application of perpendicular forces to an implant with a slight varus inclination resulted in a significantly elevated fracture load of the medial tibia, i.e., an increase of about 34% between 0° and 3° inclination. This observation suggests that inclining the implant between 0° and 3° steadily increases the fracture load to 3°, beyond which it does not significantly increase anymore.

A review of the literature suggests that varus alignment may offer a number of biomechanical advantages. Varus positioning of the tibial implant in UKAs has been demonstrated to provide a more even stress distribution across the medial compartment, which correlates with a lower risk of PPF (Wood et al., 2024; Dai et al., 2018; Inoue et al., 2016; Innocenti et al., 2016). Clinical studies have demonstrated a reduction in the risk for PPF when the tibial implant is positioned in a slight varus as a valgus alignment correlates with an increase in risk for PPF (Suda et al., 2022; Vasso et al., 2015).

The varus alignment of the tibial component in UKAs results in an increase in the distance between the keel of the tibial implant and the medial cortex of the tibia. The growth of bone between the keel and the cortex may contribute to enhanced bone stability and a reduced risk for PPF. These findings are supported by a recent retrospective clinical study which demonstrated an association between the incidence of PPF and a decreased medial keel-cortex distance in a large study sample (Watrinet et al., 2024a). Indicating that a medial keel-cortex distance has a positive influence on the incidence of PPF.

Our data show that a slight varus of 3° when introducing the force perpendicular to the implant correlates with a significantly increased fracture load by about 34%. The fracture load does not significantly increase further when applying a varus higher than 3°, indicating that the preferred placement of the tibial implant is at a slight varus. Our assumption is supported by Sekuguchi et al. (2019), who proposed a musculoskeletal computer simulation to demonstrate that a varus alignment of the tibial component exceeding 4° results in excessive translation of the medial collateral ligament. Their results show that a varus alignment of the tibial implant at 2° in the coronal plane would be preferable (Sekiguchi et al., 2019).

Interestingly, our experimental set up only showed an altered fracture load when introducing the force perpendicular to the implant. This altered fracture load could not be observed when applying force parallel to the tibial shaft axis. The configuration is designed to simulate two distinct anatomical load conditions within the context of a static load case. We hypothesize that a major player in reducing fracture risk is a dynamic distribution of compressive forces upon a sudden increase in load. When a sudden load occurs on the tibial implant (e.g., jumping, walking, etc.), the mobile meniscal bearing can slip for some time on the implant surface. During this slip, only the perpendicular forces can act on the bone, in which case we found an increased fracture toughness. After a short time, the mobile meniscal bearing will be stopped by the tension of the medial collateral ligament.

### 4.1 Statistical model

The linear relationship of the variance and the interpretation of the force displacement curves leads to the formulation of the following simplest fracture probability distribution function. This function describes the likelihood of bone survival (i.e., not breaking) upon multiple repetitions of stress and in dependency of the applied forces. The probability for survival *W* was defined as:

$$W=e^{-\frac{F-F_{0\;}}{F_{A}}\frac{t}{t_{0}}}$$ (1)

*F* is the force acting on the bone, *t* is the duration of the force application. The factors *t*_*0*_ and *F*_*A*_ are renormalizations, with *F*_*A*_ additionally representing the fracture toughness of the bone in this mode of mechanical response. F_0_ is the offset between the two regimes in the force displacement diagram. Equation 2 shows analytically that for this model and the given constant deformation velocity *v*, an analytic equation for the variance and the mean values can be derived.

$$\sigma^{2}={<F^{2}>-<F>}^{2}=\left(2-\frac{\pi }{2}\right)\;*\;A^{2}$$ (2)

*F* here is the force at fracture, <F^2^>−<F > ^2^ is the variance and *A* is a variable derived from the measurements. As typical for such stochastic models, one finds a correlation between the means squared and the variance. It follows that the mean <F> is defined as

$$<F>=\sqrt{\pi /2}\;A$$ (3)

From Equation 1, the most reasonable variable to express the variance is F_A_, therefore the, expressing the time *t* and the force *F* via the constant deformation velocity *v* and the slope in the second region of the force displacement diagram *E* Equation 4 follows:

$$A^{2}=F_{A}t_{0}E\frac{v}{2}$$ (4)

Since we found that the variance correlates linearly with *φ*, also *F*_*A*_ correlates linearly with *φ* by construction.

**FIGURE 5:**
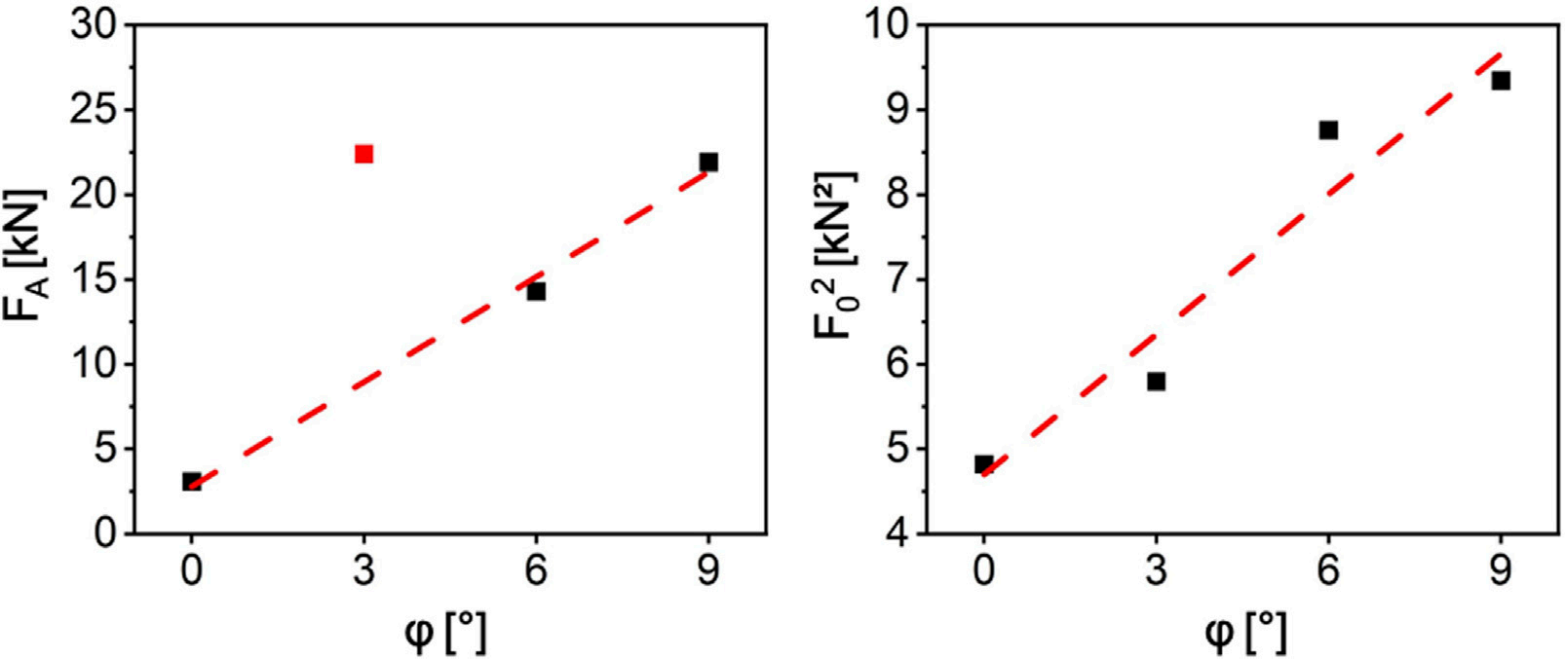
The plots of F_A_ and F_0_^2^ over the angle a. It results in two almost perfect straight lines. For the F_A_ linear fit, the data point at 3° marked in red was ignored. The reason is one outlier which has not been removed from the data.

From this variance, the theoretical mean value has been calculated using Equation 3. The difference to the measured mean fracture force has been used to calculate *F*_*0*_. The results vs *φ*, resp. F_A_ are shown in Figure 5. Not unexpectedly, yet another experimental hint on the correctness of this approach is the square root dependence of *F*_*0*_, found from the linear relation when plotting the square of the force displacement diagrams over the angles of *φ*. This means that *F*_*0*_ correlates linearly with the mean values of the fracture as well making this simple fracture model consistent. Therefore, we assume the F_A_ parameter to represent the fracture toughness of the bone for the subsequent discussion.

### 4.2 Dynamic model

**FIGURE 6:**
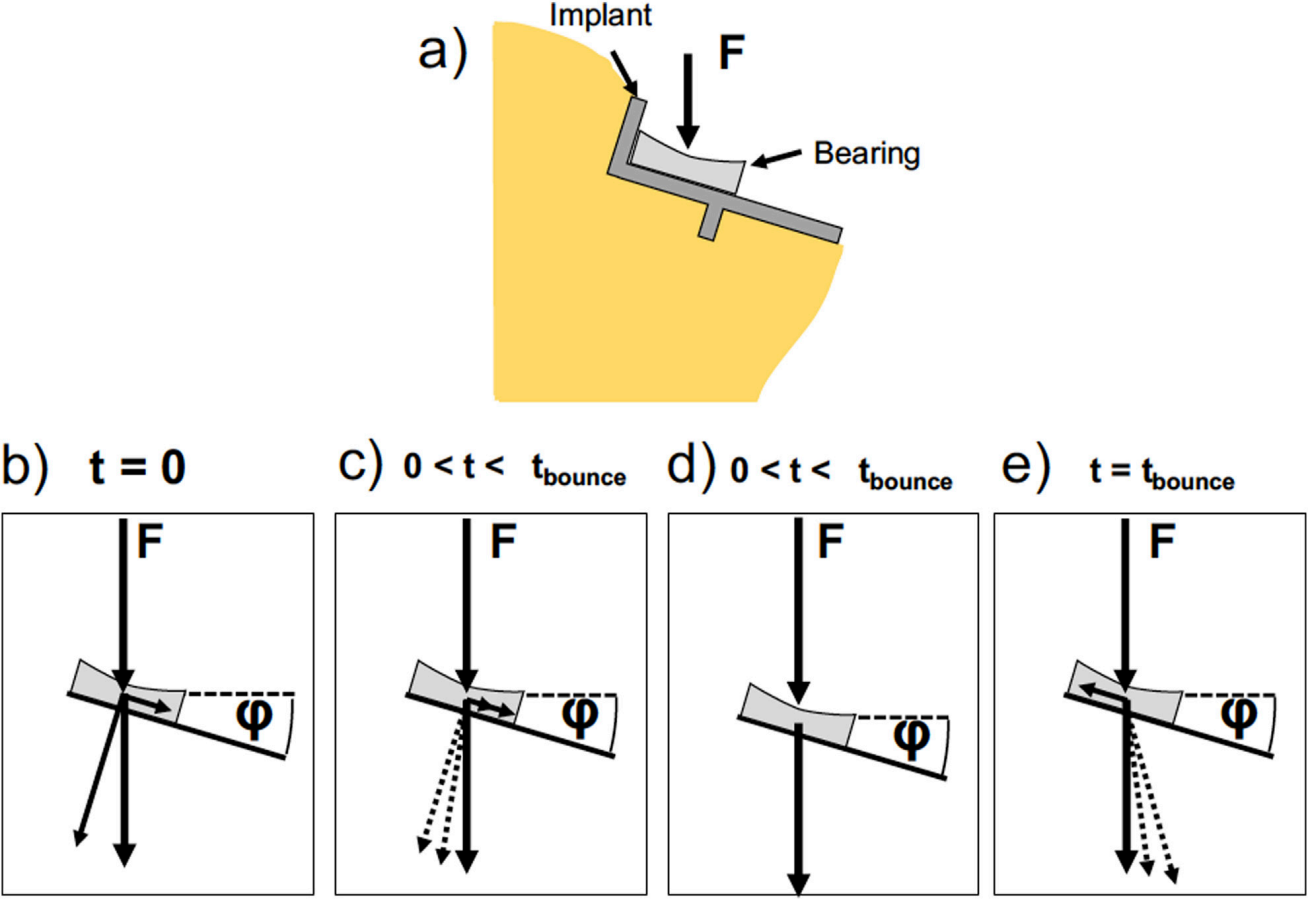
Sketch of the forces acting on the implant. **(a)** Sketch of the tibia with a tibial implant set at an angle p. The angle o is exaggerated for illustration purposes. The implant and the bearing are depicted. In the following sketches, the implant is simplified to a line for clarity. In our model we chose angles between 0 and 9°. **(b–e)** Time evolution of the forces acting on the bearing and the bone. At t = 0 **(b)** the external force acting on the bearing leads to a force component parallel to the implant surface and a force component perpendicular to the implant surface. The parallel component leads to an acceleration of the bearing, while the perpendicular force is counteracted by the bone. At 0 <t<tbounce **(c)** the bearing is accelerated and a force counteracting the parallel component is developed by the medial collateral ligament. The angle p between the external force and the counter force brought up by the bone reduces. At one point the counterforce of the tendons equals the parallel force component and the velocity is constant **(d)**. The force acting on the bone is equal to the external force. At t = tbounce **(e)** the bearing is at zero velocity and the tendons are fully stretched. The bearing is accelerated by the tendon force in the opposite direction.

This movement of the meniscal bearing in Oxford mobile bearing UKA can be modeled by coupling the properties of a spring model, i.e., linear extension with load, with a fixed length or hard stop of the extension. We used the inverse hyperbolic tangent to model this behavior and formed a simple differential equation based on the inclination angle, the static load force and the spring constants of the medial collateral ligament. The force distribution for this model is depicted in Figure 6. We apply a force for a certain duration and model the force distribution response as a consequence of it. At the beginning of the impact, the meniscal bearing is accelerated without friction or inertia so that the force is acting on the bone under the implant angle *φ.* While it is being accelerated, the collateral ligament is displaced and a counter force is developed that compensates the parallel component of the external force and reduces the angle φ under which the force is transferred to the bone.

At some point, the forces of the ligament and the parallel force component equal out and the speed of the meniscal bearing becomes constant. At this point, the force brought up by the bone is exactly equal to the external force at φ = 0. Upon further stretching of the ligament the bearing is slowed down and the ligament force exceeds the parallel force component, upon which φ becomes negative. Therefore, some of the forces acting on the bone become tensile forces, pulling on the bone. The dynamics of the system is modeled as Equation 5:

$$\ddot{x}=-\frac{D}{m}x-\frac{D}{F_{A}}\dot{x}+\frac{F_{p}}{m}$$ (5)

With the ligament’s spring constant D, the meniscal bearing’s mass m, the parallel force component Fp = F cos (φ) and the speed $\dot{x}$ and acceleration $\ddot{x}$ . The term $-\frac{D}{F_{A}}\dot{x}$ models the friction in the system and is switched on when *F*_*p*_ ≤ 0, i.e., after the ligament force becomes dominant.

Additionally, derived from Equation 1, one finds the probability density in Equation 6:

$$\dot{w}=\frac{F_{0}\left(\varphi \left(t\right)\right)-F\left(t\right)}{F_{A}\left(\varphi \left(t\right)\right)}\exp\left(t\frac{F_{0}\left(\varphi \left(t\right)\right)-F\left(t\right)}{F_{A}\left(\varphi \left(t\right)\right)}\right)$$ (6)

We have modeled the behavior by assuming linearity of *F*_*A*_ and *F*_*0*_ even to angles below 0. The duration of load application *t*_*0*_ and the constant external force *F* were used as renormalization. Therefore, the relative time is measured in units of *t*_*0*_ and *F* is selected to that it leads to fracture over the duration of *t*_*0*_ for *φ* = 0°. Additionally, we assume energy dissipation (e.g., friction) as soon as the ligament force equals the parallel force component.

**FIGURE 7:**
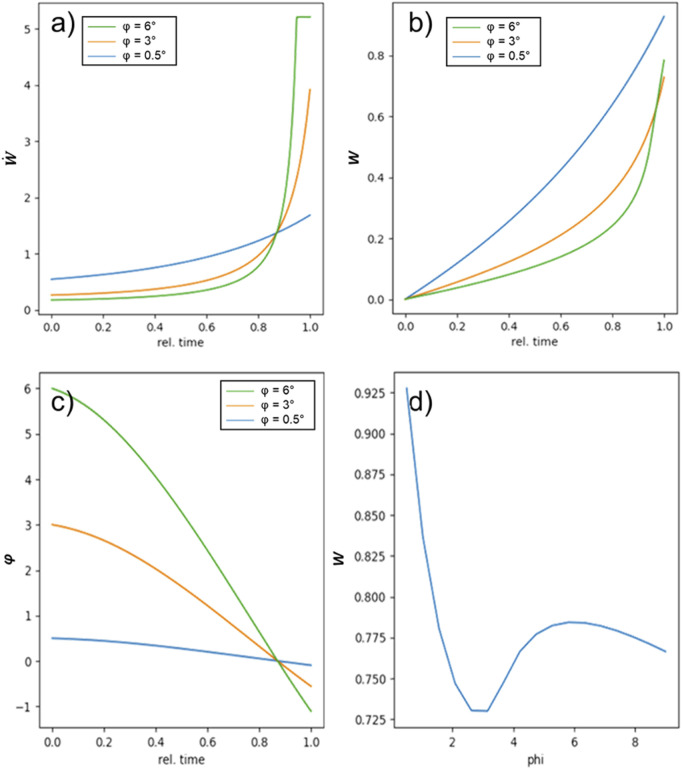
Results of the dynamic model: **(a,b)** show the probability density and the probability for three different values of p. One can see that for the higher varus inclinations, the probability density increases considerably at the end of the force pulse. In **(c)** the development of the angle at which the force is coupled into the bone develops at a consequence of counteracting ligament force development. For all varus inclinations, the angle reaches 0 at the same time, indicating constant velocity, i.e., equilibrium of all acting forces. Above this, the angle becomes negative for 3° and 6°, leading to a higher fracture probability. In **(d)** the fracture probability for the modeled experiment is depicted. Under the set of assumptions, a clear minimum in the fracture probability is found at around 3°.

Under these circumstances, the probability densities and fracture probabilities can be calculated for the various varus inclination angles *φ*, the external force *F* and the dampening, i.e., the magnitude of energy dissipation. The results of the modeling can be found in Figure 7. One finds that the overshooting of the meniscal bearing and the extension of the ligament lead to a significant increase in the fracture probability. This increase is not as pronounced for 3° and the benefits of a lower fracture probability for *φ* > 3° becomes significant. Therefore, one can find a minimum of the fracture probability at 3° varus inclination. Since the detrimental effect of the dynamics to the fracture probability increase with varus inclination and the static increase of fracture load plateaus after 3° varus inclination, the ideal tilt angle can be assumed somewhere above 0° and below 3° varus inclination.

## 5 Limitations

First, the models the models used in our study were artificial bone models. This is a potential limitation to the generalizability of our findings as the models are not capable of fully replicating the complex microarchitecture, variable density, and biological properties of authentic human bone. In particular, the absence vascularization in synthetic models can result in discrepancies in mechanical behavior and implant integration compared to native bone (Heiner, 2008). Additionally, the microstructure may have a strong bearing on the stress distributions and may completely alter the applied loads so that the dynamic model we employed is not valid. Our model did not incorporate a slope angle, which is an important factor in replicating the anatomy of the tibial plateau in human subjects. The omission of the tibial slope angle may influence joint kinematics, ligament loading, and contact forces, potentially affecting the biomechanical relevance of the findings; future research should systematically investigate how variations in tibial slope impact implant performance and knee stability in both experimental and clinical settings (Watrinet et al., 2024a; Small et al., 2013; Giffin et al., 2004; Goodfellow et al., 2011). In experimental settings it is noteworthy that joint kinematics and ligament loadings are difficult to represent faithfully as the mechanical system of the knee is complex and easily misrepresented. We suggest the use of a cadaver model for these more advanced tests. Moreover, only one implant size was utilized, which was compatible with the selected Sawbones model. As a result, the effect of undersizing or oversizing on the load distribution fracture risk was not incorporated (Watrinet et al., 2024b). We expect that only with severe undersizing the load distribution in our model and the resulting fracture results would change, as the contact area between the bone and the implant is most important for the stress distribution and this contact area does not change upon oversizing. In a real bone, however, both oversizing and undersizing have more severe effects due to the placement of the ligaments and microstructuring of the bone, respectively.

The dynamic simulation relied on significant energy dissipation after extension of the collateral ligament, which is not fully linked to respective processes in the knee. The friction of the meniscal bearing on the implant is negligible due to its material combination. The behavior of the fracture toughness for *φ* < 0 was assumed linearly but not quantified. For these and other reasons, one can say that there is some evidence of there being an optimal implant angle but it cannot be stated with sufficient certainty yet to adapt the medical procedure regulations based on this model alone.

Despite these limitations, the study provides valuable insights into the mechanical behavior of tibial components in UKA procedures and resulting fracture risk.

Replication of this study for lateral UKA is limited, as the lateral compartment has distinct anatomical and kinematic properties that substantially affect fracture mechanics and implant behavior. Therefore, our findings for medial UKA cannot be directly extrapolated to lateral UKA, and specific biomechanical studies for the lateral compartment would be needed to validate these results (Goodfellow et al., 2011).

## 6 Conclusion

Our study shows that a slight varus inclination in the frontal plane of φ < 3° is associated with a significantly reduced probability of tibial fracture under perpendicular loading. A varus inclination of 3° resulted in a 34% increase in fracture load compared to neutral alignment, with no further statistically significant increases beyond this angle. Our findings support existing literature suggesting that varus positioning optimises stress distribution and reduces the risk of PPF. In addition, our dynamic model provides insight into the biomechanical mechanisms underlying this effect. The ability of the mobile meniscal bearing to shift on impact may contribute to increased fracture toughness, particularly when the tibial component is aligned in slight varus. This highlights the importance of considering implant positioning to reduce post-operative complications.

While the results suggest that slight varus alignment is biomechanically advantageous, the practical surgical implication of whether routine implant placement in 3° varus should be considered standard requires careful evaluation in clinical settings. Further research is warranted to both confirm these findings and refine surgical recommendations. Such research should include *in vivo* analyses and studies using bone models with preserved biological properties.

## Data Availability

The original contributions presented in the study are included in the article/Supplementary Material, further inquiries can be directed to the corresponding author.
