# Supplementary figures and images for: The inclination of the tibial component has an impact on fracture stability in unicompartmental knee arthroplasty: an artificial bone study
Source: Front Bioeng Biotechnol. 2025 Oct 14;13:1615216. doi: 10.3389/fbioe.2025.1615216 (PMC12558962; doi:10.3389/fbioe.2025.1615216)

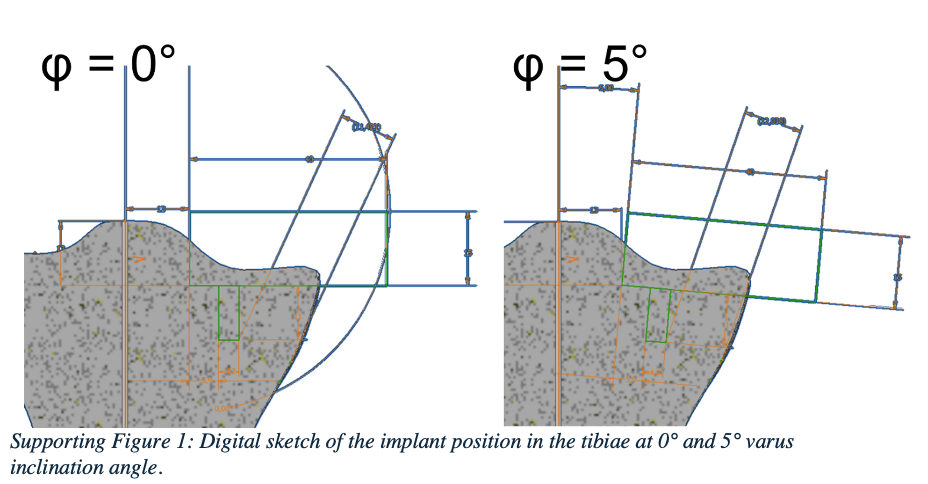

Supplement: Supplementary file 1 [file Image1.tiff]

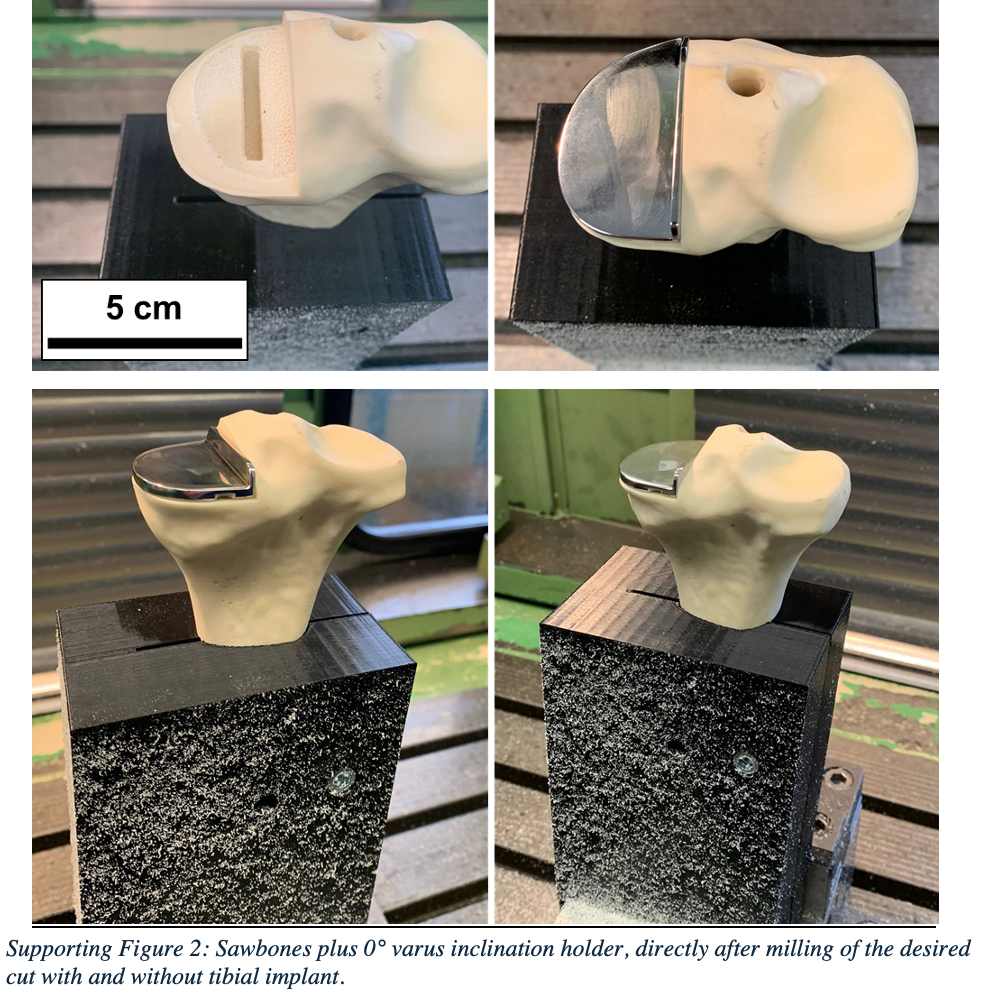

Supplement: Supplementary file 3 [file Image2.tiff]
