# Supplementary material for: The inclination of the tibial component has an impact on fracture stability in unicompartmental knee arthroplasty: an artificial bone study
Source: Front Bioeng Biotechnol. 2025 Oct 14;13:1615216. doi: 10.3389/fbioe.2025.1615216 (PMC12558962; doi:10.3389/fbioe.2025.1615216)
Supplement: Supplementary file 2 [file Supplementaryfile1.docx]

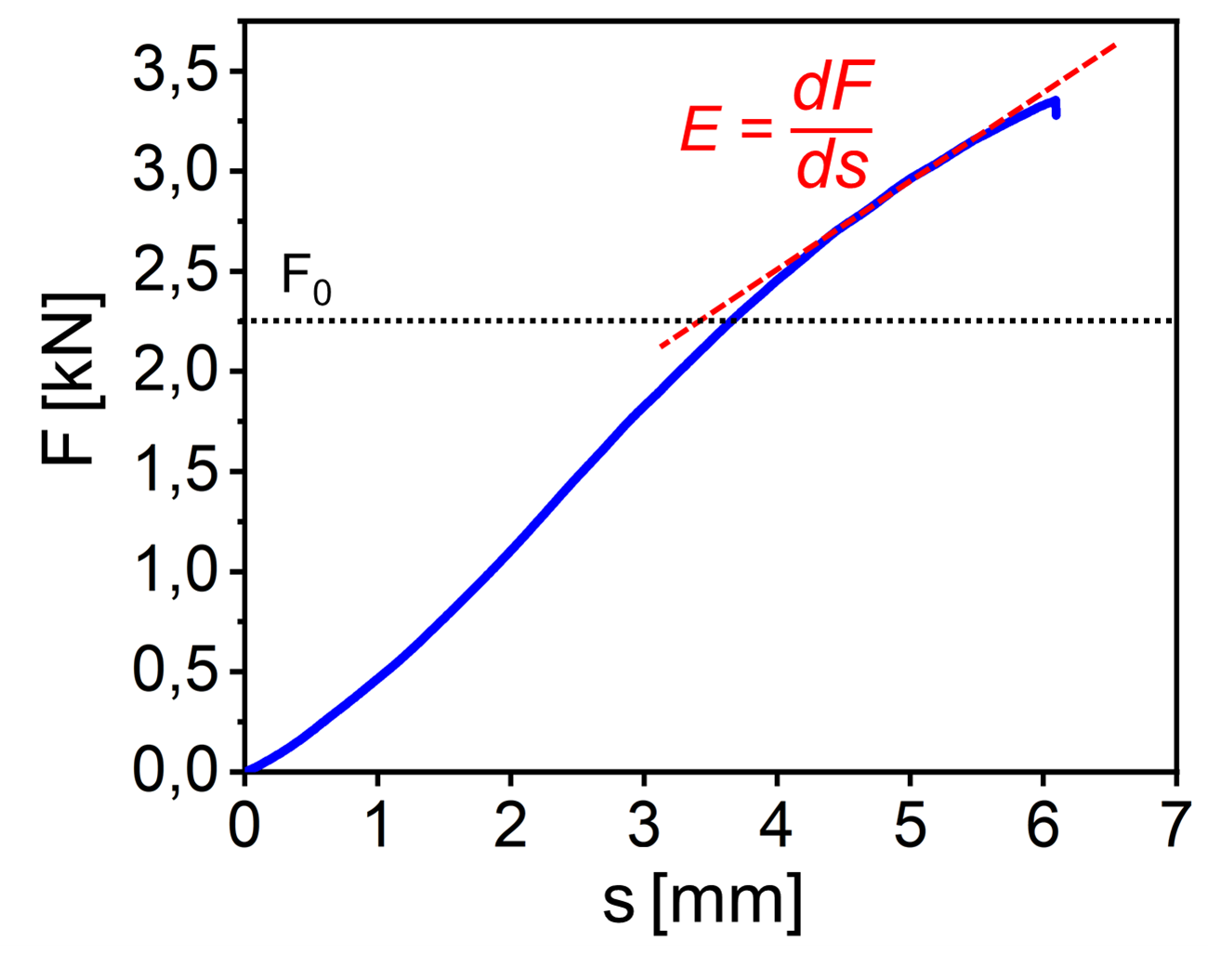


Figure 1 Exemplary force displacement curve for a tibia loaded until fracture. Two distinct linear regions can be identified. The slope of the second region is defined as E. The transition point between first and second region is defined as F_0_


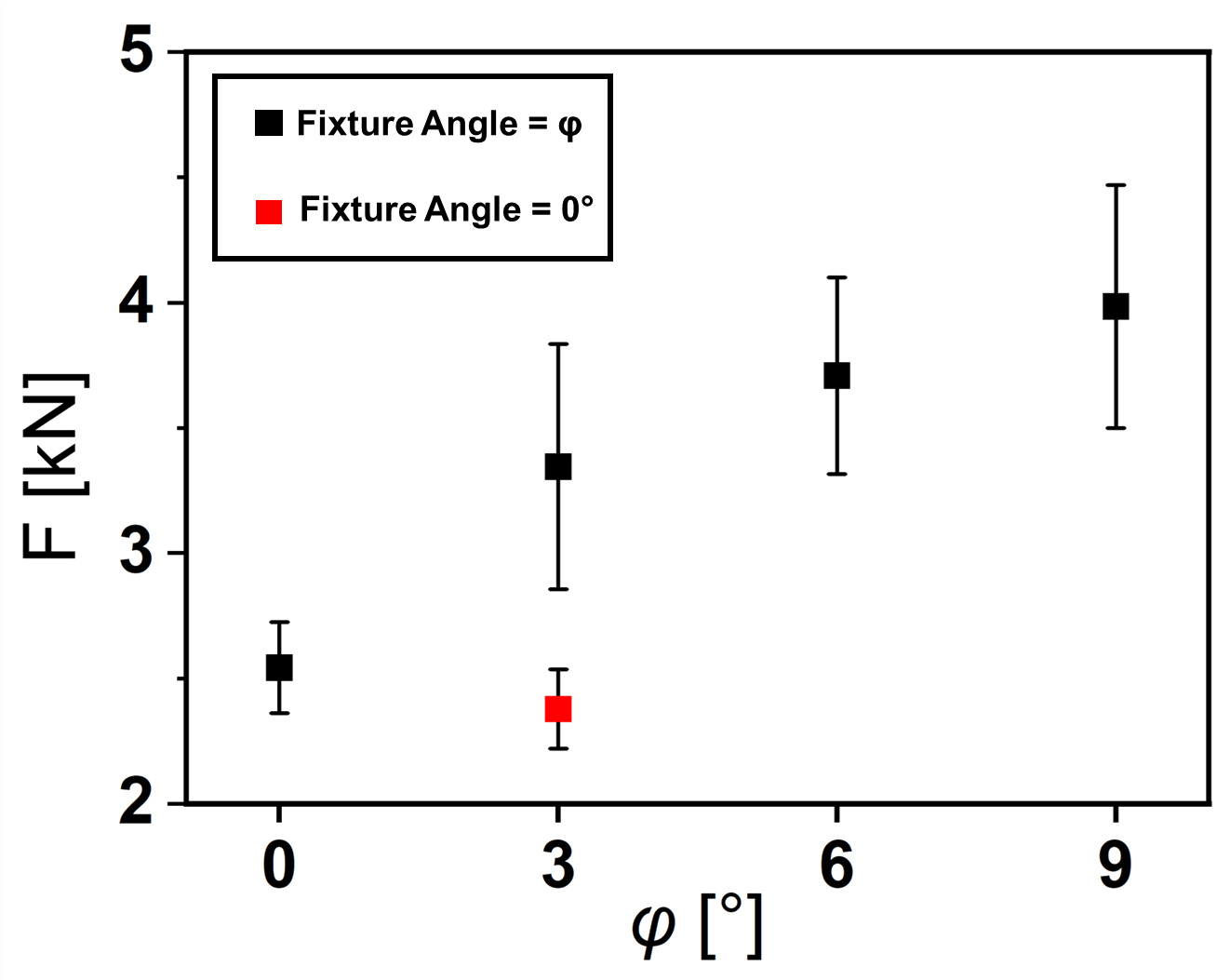


Figure 2: Fracture Loads depending on the varus inclination φ of the tibial saw cuts. In the test groups (black rectangles), forces where applied perpendicular to the implant. In the red test group, forces where applied parallel to the tibial shaft axis.


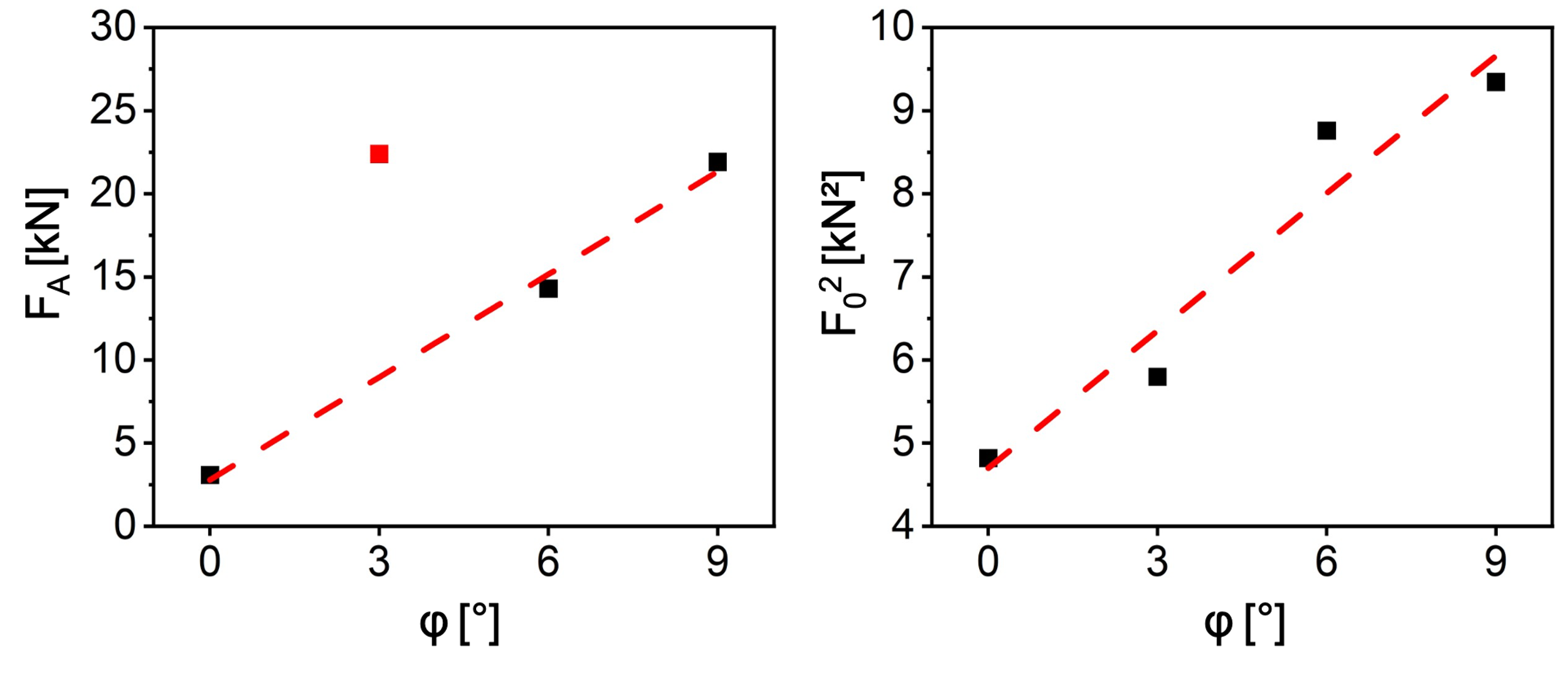


Figure 3 The plots of F_A_ and F_0_² over the angle α. It results in two almost perfect straight lines. For the F_A_ linear fit, the data point at 3° marked in red was ignored. The reason is one outlier which has not been removed from the data.


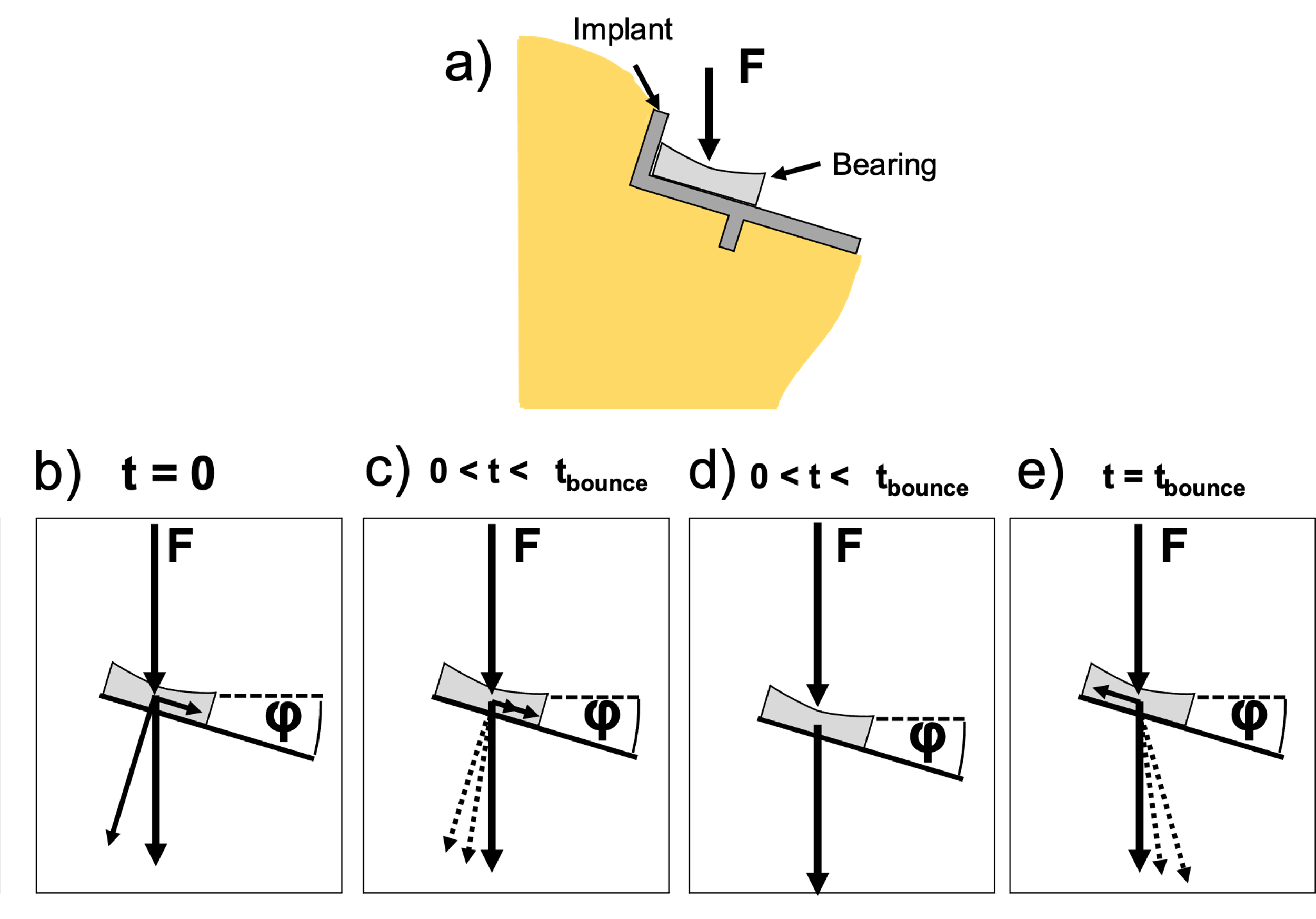


Figure 4 Sketch of the forces acting on the implant. a) Sketch of the tibia with a tibial implant set at an angle φ. The angle φ is exaggerated for illustration purposes. The implant and the bearing are depicted. In the following sketches, the implant is simplified to a line for clarity. In our model we chose angles between 0 and 9°. b-e) Time evolution of the forces acting on the bearing and the bone. At t = 0 (b)) the external force acting on the bearing leads to a force component parallel to the implant surface and a force component perpendicular to the implant surface. The parallel component leads to an acceleration of the bearing, while the perpendicular force is counteracted by the bone. At 0 < t < t_bounce_ (c)) the bearing is accelerated and a force counteracting the parallel component is developed by the medial collateral ligament. The angle φ between the external force and the counter force brought up by the bone reduces. At one point the counterforce of the tendons equals the parallel force component and the velocity is constant (d)). The force acting on the bone is equal to the external force. At t = t_bounce_ (e)) the bearing is at zero velocity and the tendons are fully stretched. The bearing is accelerated by the tendon force in the opposite direction.


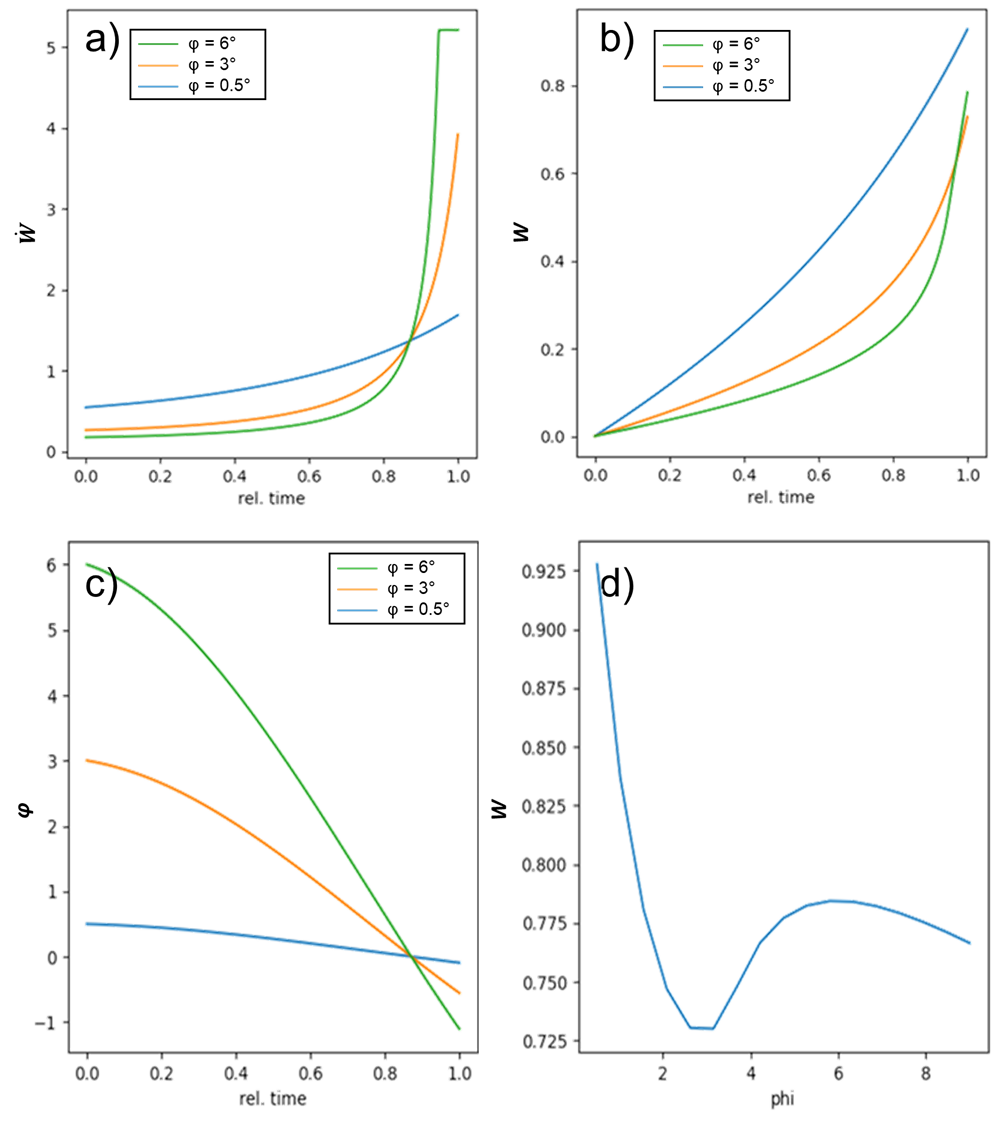


Figure 5 Results of the dynamic model: a) and b) show the probability density and the probability for three different values of φ. One can see that for the higher varus inclinations, the probability density increases considerably at the end of the force pulse. In c) the development of the angle at which the force is coupled into the bone develops at a consequence of counteracting ligament force development. For all varus inclinations, the angle reaches 0 at the same time, indicating constant velocity, i.e., equilibrium of all acting forces. Above this, the angle becomes negative for 3° and 6°, leading to a higher fracture probability. In d) the fracture probability for the modeled experiment is depicted. Under the set of assumptions, a clear minimum in the fracture probability is found at around 3°.

Supporting Figures


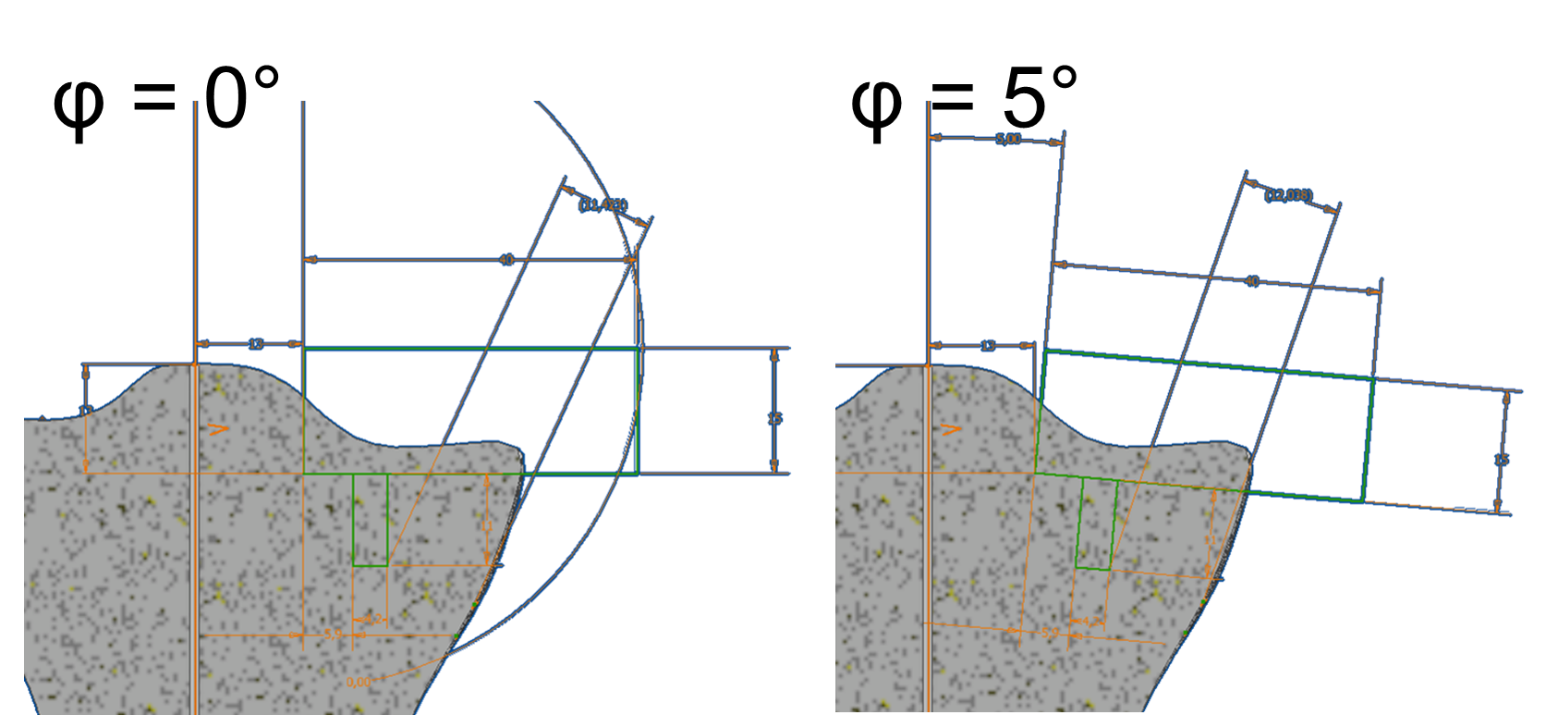


Supporting Figure 1 Digital sketch of the implant position in the tibiae at 0° and 5° varus inclination angle.


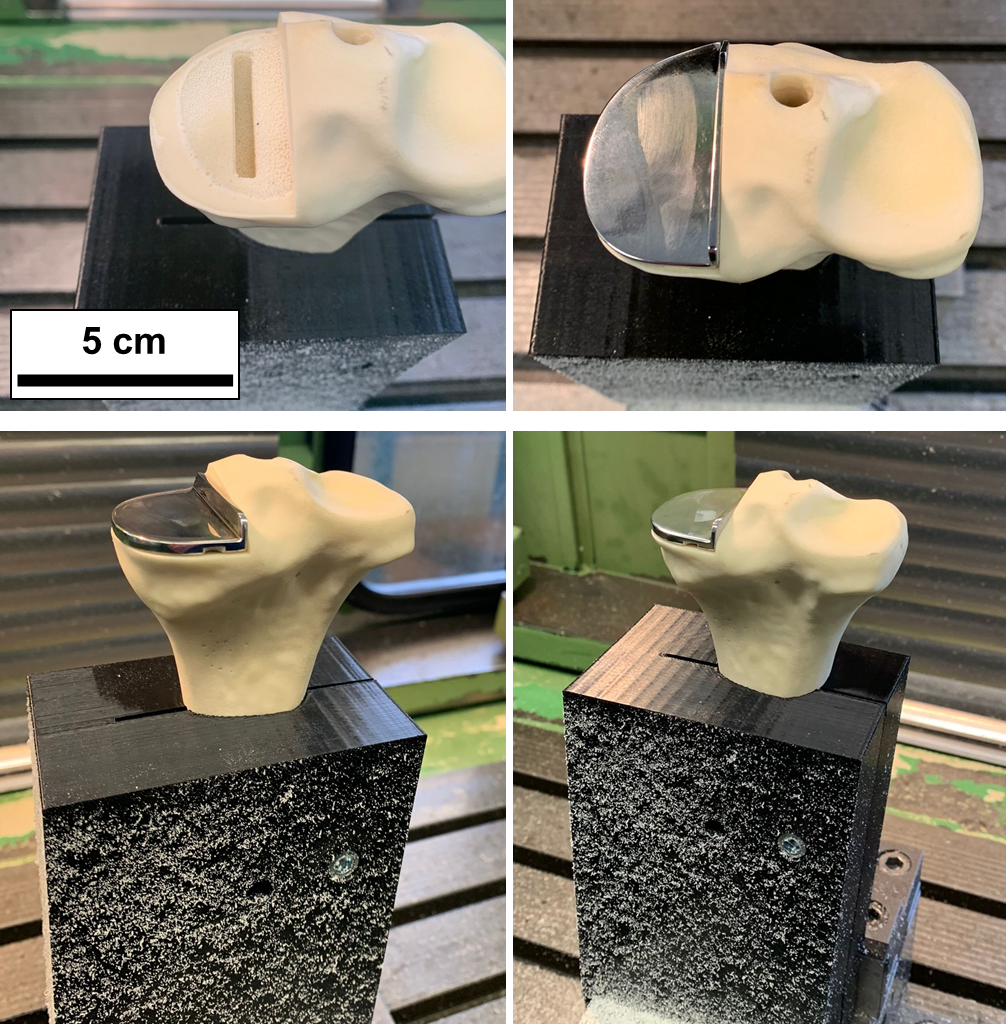

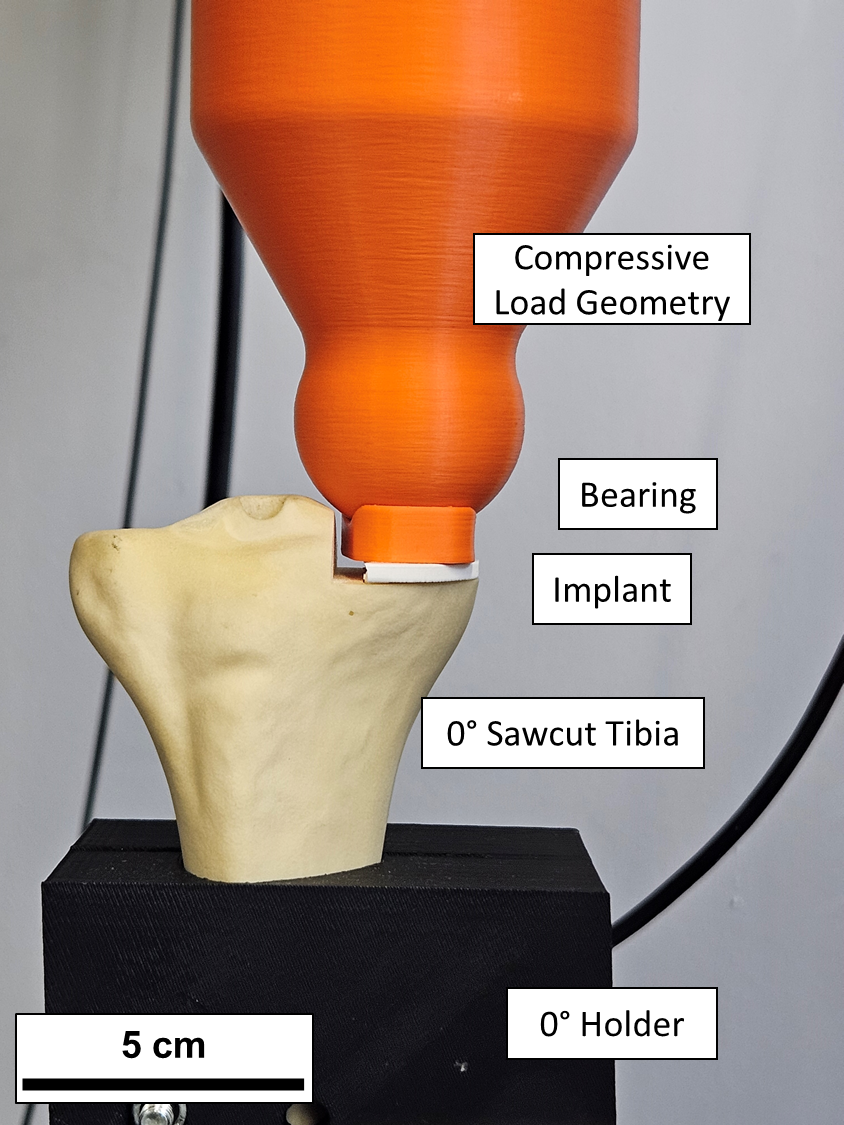


Supporting Figure 2 Sawbones plus 0° varus inclination holder, directly after milling of the desired cut with and without tibial implant.

Supporting Figure 3 Sawcut tibia bone with implant and bearing in the compression test apparatus. The implant and meniscal bearing in this instance were 3D printed for setup validation and for the real tests replaced with originals.


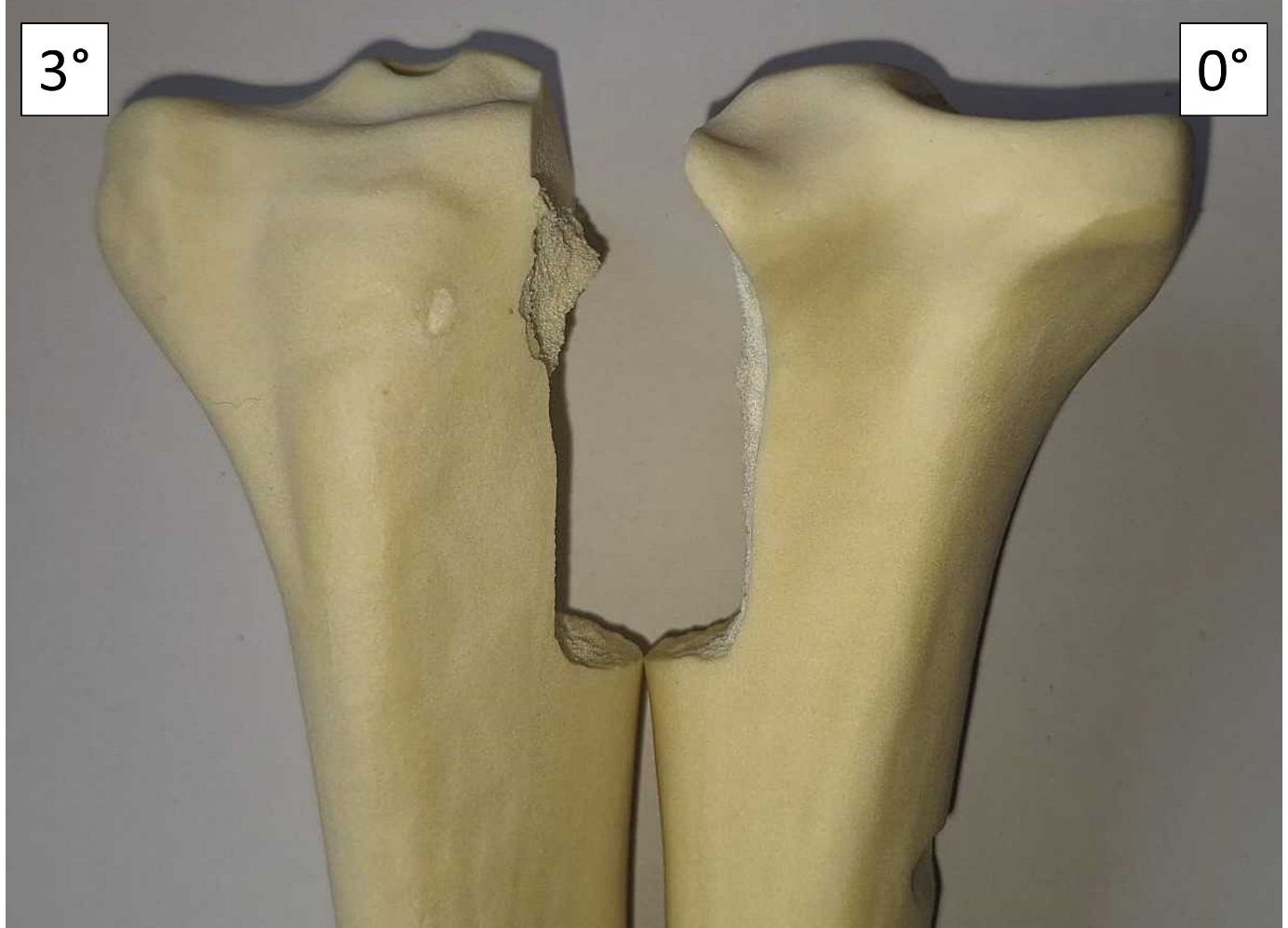


Supporting Figure 4 Fracture profiles of the tibiae with 3° and 0° varus inclination. At 3° inclination, the fracture becomes more complex and the fracture area is increased.
